# Multi-center evaluation of the Research Use Only NeuMoDx monkeypox virus (MPXV) fully automated real-time PCR assay

**DOI:** 10.1128/jcm.00028-24

**Published:** 2024-04-19

**Authors:** Heba H. Mostafa, Gavin Wall, Szu-Chi Su, Gerta Hysa, Lijie Gong, Urbain Charly Dadjeu, Helen Cheung, Andrew Pekosz, Delphine De Smet, Nikola Sklenovská, Lies Laenen, Laura Viñuela, Adolfo de Salazar, Ana Fuentes, Elizaveta Padalko, Federico Garcia

**Affiliations:** 1 Division of Medical Microbiology, Department of Pathology, Johns Hopkins School of Medicine, Baltimore, Maryland, USA; 2 QIAGEN GmbH, Hilden, Germany; 3 NeuMoDx Molecular Inc, a QIAGEN company, Ann Arbor, Michigan, USA; 4 W. Harry Feinstone Department of Molecular Microbiology and Immunology, The Johns Hopkins Bloomberg School of Public Health, Baltimore, Maryland, USA; 5 Universitair Ziekenhuis Gent, Gent, Belgium; 6 Laboratory of Clinical and Epidemiological, Virology, Department of Microbiology, Immunology and Transplantation, Rega Institute, KU Leuven, Leuven, Belgium; 7 Department of Laboratory Medicine, University Hospitals Leuven, Leuven, Belgium; 8 Laboratory of Clinical Microbiology, Department of Microbiology, Immunology and Transplantation, KU Leuven, Leuven, Belgium; 9 Hospital Universitario Clínico San Cecilio, Granada, Spain; 10 Instituto de Investigación Biosanitaria IBS.GRANADA, Granada, Spain; 11 Ciber de Enfermedades Infecciosas, CIBERINFEC, Madrid, Spain; St. Jude Children's Research Hospital, Memphis, Tennessee, USA

**Keywords:** monkeypox virus (MPXV), real-time PCR, mpox, NeuMoDx

## Abstract

The mpox outbreak, caused by monkeypox virus (MPXV), accelerated the development of molecular diagnostics. In this study, we detail the evaluation of the Research Use Only (RUO) NeuMoDx MPXV assay by multiple European and US sites. The assay was designed and developed by Qiagen for the NeuMoDx Molecular Systems. Primers and probes were tested for specificity and inclusivity *in silico*. The analytical sensitivity of the assay was determined by testing dilutions of synthetic and genomic MPXV DNA. A total of 296 clinical samples were tested by three sites; the Johns Hopkins University (US), UZ Gent (Belgium, Europe), and Hospital Universitario San Cecilio (Spain, Europe). The analytical sensitivity of the assay was 50 copies/mL for both clades I and II. The assay showed 100% *in silico* identity for 80 clade I and 99.98% *in silico* identity for 5,162 clade II genomes. Clade II primers and probes showed 100% *in silico* specificity; however, identity of at least one of the two sets of clade I primers and probes with variola, cowpox, camelpox, and vaccinia viruses was noticed. The clinical validation showed sensitivity of 99.21% [95% confidence interval (CI): 95.66–99.98%] and specificity of 96.64% (95% CI: 91.62–99.08%) for lesion swab samples. The NeuMoDx MPXV Test shows acceptable analytical and clinical performance. The assay improves the laboratory’s workflow as it consolidates nucleic acid extraction, PCR, data analysis, and interpretation and can be interfaced. The Test Strip can differentiate clades I and II, which has important laboratory safety implications.

**IMPORTANCE:**

In this manuscript, we provide detailed *in silico* analysis and clinical evaluation of the assay using a large cohort of clinical samples across three academic centers in Europe and the United States. Because the assay differentiates MPXV clades I and II, this manuscript is timely due to the current need to rule out the regulated clade I by diagnostic clinical laboratories. In December 2023, and due to first report of cases of sexually transmitted clade I infections in the Democratic Republic of the Congo, when generic assays that do not differentiate the clades are used, samples are considered regulated. The assay meets the need of full automation and has a marked positive impact on the laboratory workflow.

## INTRODUCTION

The 2022 outbreak of mpox, the disease caused by monkeypox virus (MPXV) was yet another challenge for the clinical diagnostic laboratories shortly after the huge burden of the COVID-19 pandemic. MPXV is a member of the *Orthopoxvirus* genus, family *Poxviridae*, and its first detection from humans was in 1970 (1–3). Until before the outbreak of 2022, the disease was endemic in certain African countries with occasional outbreaks outside the areas of endemicity, including an outbreak that was related to close contact with prairie dogs (4) and travel-related cases (5, 6). In May 2022, multiple cases, with no travel history to endemic regions were reported from multiple countries and the global cases as of 30 November 2023 reached 92,167 (7).

Prompt validations and implementation of assays for the detection of mpox cases were essential for prompt diagnosis and infection control. Laboratories quickly implemented testing using laboratory developed assays that were MPXV-specific or based on the CDC non-variola orthopoxvirus assay (8–10). Two primary challenges and two main needs were encountered by the laboratories upon validating and implementing mpox molecular assays: (i) difficulty obtaining control materials, (ii) inaccessibility to clinical samples, (iii) the need for automated solutions to reduce errors and handle large volume testing, and (iv) the need for differentiating MPXV clade I from clade II for biosafety related purposes. In particular, differentiating clades I and II (previously Congo Basin and Western African clades, respectively) has been required by laboratories testing for mpox. All infections during the 2022–2023 outbreak were caused by clade II. Clade I samples are classified and regulated as select agents. In December 2023, and due to the first report of cases of sexually transmitted clade I infections in the Democratic Republic of the Congo, when generic assays that do not differentiate the clades are used, samples are considered regulated (11, 12).

In this study, a research-use-only, dual-target real-time PCR assay that detects and differentiates MPXV clades I and II DNA from lesion swabs was developed for the random-access, fully automated, NeuMoDx Molecular systems (QIAGEN GmbH, Hilden, Germany). The assay was developed and analytically validated by Qiagen (using *in silico* and control materials) and was evaluated for clinical use by three independent sites in Europe and the United States (using a large cohort of clinical specimens).

## MATERIALS AND METHODS

### The NeuMoDx MPXV assay (RUO)

The qualitative real-time PCR test, the NeuMoDx MPXV Assay, detects dual targets for clades I and II of MPXV in two separate channels. Clade I targets are C3L and B21R genes, and Clade II targets are G2R (TNF receptor) and OPG005 genes. Targets were based on the Qiagen GeneGlobe design. A third channel detects an internal control (spc2), that controls for sample inhibition. The assay is fully automated and integrates sample extraction, real-time PCR, results interpretation, and reporting.

### Limit of detection

Limit of detection (LOD) was characterized using quantified synthetic/genomic DNA: Clade II genomic (Monkeypox Virus, USA-2003, Thermo Fisher) and synthetic DNA (BEI Resources), and Clade I synthetic DNA (Metabion, quantified by Qiagen using the QIAcuity digital PCR system). Genomic materials were used to make dilutions in transport medium [VTM (Sigma, Virocult) and UVT (BD)].

### Inclusivity

*In silico* analysis of 5,248 available MPXV sequences in Global Initiative on Sharing Avian Influenza Data (GISAID, 2659) and National Center for Biotechnology Information (NCBI, 2589) was performed (clade II: 5,168 and clade I: 80 genomes). The NCBI’s Basic Local Alignment Search Tool (BLAST) was used to analyze the sequences. Complete high-quality genomes deposited as of November 2022 (>196,000 bp) were used for the analysis (genomes with missing nucleotides or with ambiguous or degenerate bases were considered invalid and were excluded, for clade II, six invalid sequences were excluded due to suboptimal coverage of the primers and probe binding sites of the NeuMoDx MPXV assay. Samples were categorized as perfect match (0 mismatch, no mismatch across all primers and probe sequences), one mismatch (sequences where at least one of the primers or probe sequences had only one nucleotide mismatch with no sequences having two or more mismatches), or 2+ mismatches (sequences where at least one of the primers or probe sequences have two or more nucleotide mismatches).

Analytical specificity was demonstrated through an *in silico* study of 33 pathogens, including six from the *Orthopoxvirus* genus. BLAST searches using the NeuMoDx MPXV primer and probe sets against NCBI nucleotide database (NCBI database, as of 17 October 2022) were performed. Analysis included only sequences with full coverage of all three oligonucleotide-binding regions for each of the four primer/probe sets. Cross-reactivity was predicted when the sequence analysis revealed ≥85% match to both primers and the probe in the two primer and probe sets for each respective clade. Wet laboratory testing of clinical samples positive for HSV-1, HSV-2, and VZV by standard-of-care testing at the Johns Hopkins and UZ Gent, in addition to vaccinia, and cowpox Quality Control for Molecular Diagnostics (QCMD) and virus stock samples [research strains (13, 14)] was performed. Additionally, UZ Gent performed testing for monkeypox/orthopoxvirus panels (418, INSTAND eV (lyophilized cell culture supernatants) and Pox 2023, QCMD).

### Standard-of-care (reference) testing

Standard-of-care testing at the Johns Hopkins Medical Microbiology Laboratory was performed using a laboratory developed orthopoxvirus assay (8), originally developed by the CDC for the detection of the E9L DNA polymerase gene of non-variola orthopoxviruses (15). DNA extraction was performed using the NucliSENS easyMag or eMag instruments (Biomerieux, Marcy-l’Étoile, France) using software version 2.1.0 following an off-board lysis manufacturer’s protocol. A total of 500 or 250 µL samples were extracted and eluted in 50 or 25 µL. Real-time PCR was performed using the ABI Prism 7500 Sequence Detection System (Applied Biosystems) as previously described (8).

The Belgian (BE) standard-of-care testing was performed using a laboratory developed MPXV assay. Samples were inactivated at 70°C for 5 min before extraction. Total nucleic acid extraction was performed using the NucliSENS easyMAG or eMAG (Biomerieux, Marcy-l’Étoile, France) with an input volume of 220 µL and elution in 110 µL. The generic protocol was used after off-board lysis for 10 min with continuous shaking. Phocine Herpesvirus was added as a DNA internal control. Real-time PCR was based on a previously developed assay (16) with the following primers and probes: O2L—gene: forward: 5′-CAATAGTGAGTTCGGCGACAAAG-3′, reverse: 5′-TTGTATCGCATCTCTCGGGTATTC-3′, probe: FAM-5′-CCAGTACCGGTAATCT-3′-MGB, F3L-gene: forward: 5′-CATCATCTATTATAGCATCAGCATCAGA-3′, reverse: 5′-CGATACTCCTCCTCGTTGGTCTAC-3′, probe: NED-5′-CTGATACACGGCCTACAG-3′-MGB and was performed using a QuantStudio Dx thermocycler (Thermo Fisher Scientific).

Testing at the Spanish (ES) site was performed using either the Mpox Realtime PCR Kit (Vircell, SL) or Viasure Mpox Assay (Certest Biotec, SL) on CFX96 thermocycler. Samples were inactivated at 70°C for 5 min before extraction using the TANBead (Taiwan Advanced Nanotech Inc).

## RESULTS

### The NeuMoDx MPXV assay analytical sensitivity (LOD)

Preliminary analytical sensitivity or lower limit of detection was evaluated by testing three different dilutions of the monkeypox virus clades I and II genomic materials to determine the concentration at which 100% of the replicates are detected (Table 1). Eight replicates were tested for each dilution. This preliminary analysis showed that the LOD of the assay for detecting clades I and II was 50 copies/mL. Additional replicates were tested to confirm the analytical sensitivity for both clades by testing a minimum of 24 replicates. This follow-up experiment confirmed the LOD of the assay as 50 copies/mL for both clades I and II in two different transport media (Table 2).

**TABLE 1:** Preliminary analytical sensitivity of the NeuMoDx MPXV assay for clades I and II^*a*^

Target/material	Concentration (copies/ mL)	Number of valid results/total tested	Number of positives	% Detection	Average Ct	Ct SD
Clade I, Synthetic DNA	150	8/8	8	100%	32.48	0.35
	50	8/8	8	100%	33.83	0.50
	17	7/8	5	71%	33.75	0.46
Clade II, Genomic DNA	50	8/8	8	100%	34.01	0.29
	17	8/8	4	50%	34.37	0.32
	5.7	8/8	1	13%	34.01	N/A^*b*^
Clade II, Synthetic DNA	150	8/8	8	100%	33.19	0.37
	50	8/8	8	100%	33.87	0.45
	17	8/8	7	88%	34.21	0.56

^*a*^ Ct, cycle threshold; SD, standard deviation.

^*b*^ Not applicable.

**TABLE 2:** Analytical sensitivity of the NeuMoDx MPXV assay for clades I and II^*a*^

Target/strain	Concentration (copies/mL)	Matrix	Valid results/total tested	Number of positives	% Positives	Average Ct	Ct SD
Clade I, Synthetic DNA	50 copies/mL	UVT	22/24	22	100%	34.17	0.77
	50 copies/mL	VTM	24/24	24	100%	33.41	0.35
Clade II, Genomic DNA	50 copies/mL	UVT	23/24	23	100%	34.43	0.75
	50 copies/mL	VTM	24/24	24	100%	33.96	0.60
Clade II, Synthetic DNA	50 copies/mL	UVT	24/24	24	100%	34.00	0.72

^*a*^ Ct, cycle threshold; SD, standard deviation.

### The NeuMoDx MPXV assay inclusivity (*in silico*)

An inclusivity analysis of the NeuMoDx MPXV assay primers and probes was performed by aligning their sequences to complete monkeypox virus clades I and II genomes deposited in GISAID or NCBI databases as of November 2022. The dual target PCR primers and probe sets that were designed for clade I detection showed 100% identity to all 80 sequences analyzed (Table 3). Dual target PCR primers and probe sets that were designed for clade II detection showed 99.98% identity to the 5,162 genomes analyzed (Table 3).

**TABLE 3:** Inclusivity of the NeuMoDx MPXV assay for clades I and II

Mismatch analysis	Clade I					
80 samples	Target 1		Target 2		Coverage (NCBI + GISAID)	
Invalid	0	0%	0	0%	0	0%
Valid sequences	80		80		80	
Perfect match	80	100%	78	97.5%	80	100%
1 mismatch	0	0%	2	2.50%	0	0%
2+ mismatch	0	0%	0	0%	0	0%

Mismatch analysis	Clade II					
5,168 samples	Target 1		Target 2		Coverage (NCBI + GISAID)	
Invalid	149	2.88%	143	2.77%	6	0.12%
Valid sequences	5,019		5,025		5,162	
Perfect match	5,015	99.92%	4,992	99.34%	5,161	99.98%
1 mismatch	2	0.04%	32	0.64%	0	0.00%
2+ mismatch	2	0.04%	1	0.02%	1	0.02%

### Analytical specificity of the NeuMoDx MPXV assay

*In silico* analysis of the primer and probes biding sites with pathogens that can cause skin lesions, including HSV-1, HSV-2, and VZV as well as frequently encountered bacteria (non-phylogenetically related species likely to be found in skin lesion specimens, Table 4) showed no cross-reactivity. However, potential cross-reactivity was observed for MPXV clade I primer/probe sets with cowpox, camelpox, mousepox, vaccinia, and variola viruses. Testing left-over clinical samples positive for HSV-1, HSV-2, or VZV by standard-of-care clinical testing at Johns Hopkins and UZ Gent in addition to vaccinia and cowpox control materials or viral stocks (Table 5) was performed. No cross-reactivity was observed for all tested samples. Monkeypox and orthopoxvirus control panels tested by UZ Gent also showed 100% agreement when tested with the NeuMoDx MPXV assay (Table 5).

**TABLE 4:** Analytical specificity of the NeuMoDx MPXV assay (*in silico* analysis)

Pathogen	Number of sequences evaluated	Host(s)	Sequences with ≥85% match to primer-probe sets			
			Set 1 clade I	Set 2 clade II	Set 3 clade I	Set 4 clade II
Variola virus (smallpox)	56	Human	56	0	0	0
HSV-1	19	Human	0	0	0	0
HSV-2	20	Human	0	0	0	0
VZV	2	Human	0	0	0	0
*Staphylococcus aureus*	2,188	Human	0	0	0	0
*Streptococcus pyogenes*	512	Human	0	0	0	0
*Pseudomonas aeruginosa*	985	Human	0	0	0	0
*Corynebacterium jeikeium*	8	Human	0	0	0	0
Human genomic DNA	2	Human	0	0	0	0
*Escherichia coli*	5,789	Human	0	0	0	0
*Bacteroides fragilis*	109	Human	0	0	0	0
Cowpox virus	87	Human	80	0	4	0
Camelpox virus	10	Human	10	0	0	0
*Streptococcus* group G	103	Human	0	0	0	0
*Neisseria gonorrhoeae*	257	Human	0	0	0	0
*Mycoplasma pneumoniae*	160	Human	0	0	0	0
HPV	9,116	Human	0	0	0	0
*Treponema pallidum*	18	Human	0	0	0	0
Molluscum contagiosum virus	31	Human	0	0	0	0
Vaccinia virus	130	Human	115	0	77	0
*Streptococcus mitis*	19	Human	0	0	0	0
*Staphylococcus epidermidis*	296	Human	0	0	0	0
*Streptococcus agalactiae*	280	Human	0	0	0	0
*Trichophyton rubrum*	2	Human	0	0	0	0
*Candida albicans*	10	Human	0	0	0	0
*Lactobacilllus* species	331	Human	0	0	0	0
*Acinetobacter calcoaceticus*	10	Human	0	0	0	0
*Enterococcus faecalis*	922	Human	0	0	0	0
Ectromelia (mousepox) virus	5	Human	0	0	5	0
*Streptococcus* group C	512	Human	0	0	0	0
*Corynebacterium diphtheriae*	122	Human	0	0	0	0
*Chlamydia trachomatis*	482	Human	0	0	0	0
*Mycoplasma genitalium*	10	Human	0	0	0	0
*Trichomonas vaginalis*	4	Human	0	0	0	0

**TABLE 5:** Analytical specificity and agreement of the NeuMoDx MPXV assay

Virus	Samples tested	NeuMoDx result	Comment
HSV-1	4	Clade I/II DNA not detected	Clinical swabs
HSV-2	6	Clade I/II DNA not detected	Clinical swabs
VZV	5	Clade I/II DNA not detected	Clinical swabs
Vaccinia virus	3	Clade I/II DNA not detected	×1 QCMD sample, ×2 NIST culture fluid
Cowpox virus	1	Clade I/II DNA not detected	×1 QCMD sample
Negative		Clade I/II DNA not detected	
Clade II positive		Clade II DNA detected	
Clade II positive		Clade II DNA detected	
Negative		Clade I/II DNA not detected	
Pox 2023 panel (QCMD)			
Clade I/II		Clade I/II DNA detected	
Negative		Clade I/II DNA not detected	
Clade IIb		Clade II DNA detected	
Clade I/II		Clade I/II DNA detected	
Clade I/II		Clade I/II DNA detected	
Vaccinia		Clade I/II DNA not detected	
Clade I/II		Clade I/II DNA detected	
Cowpox		Clade I/II DNA not detected	
Clade IIb		Clade II DNA detected	
Clade IIb		Clade II DNA detected	

### Clinical testing

Testing of monkeypox-positive and -negative samples after the standard-of-care in three different sites was performed (Table 6 details the standard-of-care testing performed in each site). A total of 296 unique patients’ samples were tested by the three sites which included 252 swabs from skin or mucocutaneous lesions, 16 rectal swabs, 25 throat (pharyngeal) swabs, and 3 urine samples (Table 7). A total of eight samples had error codes [unresolved (most likely sample inhibition) or no result (results processing errors or sample detection errors)] and were excluded from the analysis (Table 7). The assay showed an overall sensitivity of 99.36% [95% confidence interval (CI): 96.48–99.98%] and specificity of 96.97% (95% CI: 92.42–99.17%) (Table 8). When the analysis was limited to swabs from skin or mucocutaneous lesions, the assay showed sensitivity of 99.21% (95% CI: 95.66–99.98%) and specificity of 96.64% (95% CI: 91.62–99.08%) (Table 8). The four false positive results had Ct values of 33–34 and two of them repeated negative by the NeuMoDx MPXV assay (notably, all four patients were considered high risk). The one false-negative result had a Ct value of 36.96 by the reference method. Positive samples by the NeuMoDx MPXV assay had a range of Ct values from 11 to 34 by the standard-of-care method.

**TABLE 6:** Standard-of-care monkeypoxvirus testing in each study site

	Johns Hopkins University (US)	UZ Gent (BE)	Hospital Universitario San Cecilio (ES)
Standard-of-care reference method	Non-variola orthopox Generic Real-Time PCR Test (CDC)	MPXV Generic Real-Time PCR Test (CDC)	Mpox Realtime PCR Kit (Vircell, SL) or 2) Viasure Mpox Assay (Certest Biotec, SL)
Sample collection range	June–December 2022 (pilot batch)	June–November 2022 (prototype batch), December 2022 (pilot batch)	June–September 2022 (prototype batch), December 2022 (Pilot batch)
Nucleic acid extraction	eMAG or easyMAG (biomerieux). 500 µL sample and 50 µL elute volume	eMAG or easyMAG (biomerieux). 220 µL sample and 110 µL elute volume	TANBead (Taiwan Advanced Nanotech Inc). 300 µL sample and 50–80 µL elute volume
Sample inactivation	No	Heat inactivation (70°C for 5 min) or lysis buffer	Heat inactivation (70°C for 5 min)
Sample types	Swabs from skin lesions collected in Universal Viral Transport Medium	Swabs from skin lesions collected in Universal Transport Medium or liquid Amies (in addition, 3 urine samples, 15 rectal, and 23 throat swabs were tested)	Swabs from skin lesions collected in Universal Transport Medium (in addition, two pharyngeal and one rectal swabs were tested)

**TABLE 7:** Clinical samples used for the study^*a*^

	Johns Hopkins University (US)				UZ Gent (BE)				Hospital Universitario San Cecilio (ES)			
Vesicle lesion swabs		NeuMoDx positives	NeuMoDx negatives	Errors		NeuMoDx positives	NeuMoDx negatives	Errors		NeuMoDx positives	NeuMoDx negatives	Errors
Positives	40	38	1	1	33	32	0	1	56	55	0	1
Negatives	30	0	30	0	49	3	43	3	44	1	42	1
Rectal swabs												
Positives	0	N/A	N/A	N/A	13	12	0	1	1	1	0	0
Negatives	0	N/A	N/A	N/A	2	0	2	0	0	N/A	N/A	N/A
Throat swabs												
Positives	0	N/A	N/A	N/A	14	14	0	0	2	2	0	0
Negatives	0	N/A	N/A	N/A	9	0	9	0	0	N/A	N/A	N/A
Urine												
Positives	0	N/A	N/A	N/A	1	1	0	0	0	N/A	N/A	N/A
Negatives	0	N/A	N/A	N/A	2	0	2	0	0	N/A	N/A	N/A

^*a*^ Not applicable.

**TABLE 8:** Performance of the NeuMoDx MPXV assay

	All sample types	Reference test (gold standard)	
		Positives	Negatives
NeuMoDx	Positives	155	4
	Negatives	1	128
	Statistic	Value	95% CI
	Sensitivity	99.36%	96.48–99.98%
	Specificity	96.97%	92.42–99.17%
	Positive likelihood ratio	32.79	12.49–86.07
	Negative likelihood ratio	0.01	0.00–0.05
	Positive predictive value	97.48%	93.66–99.03%
	Negative predictive value	99.22%	94.78–99.89%
	Accuracy	98.26%	96.00–99.43%

	Lesion swabs	Reference test (gold standard)	
		Positives	Negatives
	Positives	125	4
NeuMoDx	Negatives	1	115
	Statistic	Value	95% CI
	Sensitivity	99.21%	95.66–99.98%
	Specificity	96.64%	91.62–99.08%
	Positive likelihood ratio	29.51	11.26–77.35
	Negative likelihood ratio	0.01	0.00–0.06
	Positive predictive value	96.90%	92.26–98.79%
	Negative predictive value	99.14%	94.23–99.88%
	Accuracy	97.96%	95.30–99.33%

## DISCUSSION

The quick implementation of molecular diagnostics for mpox was essential with the beginning of the 2022 outbreak to ensure early detection, patients’ management, and infection control. Different laboratories developed assays for testing lesion swabs, the only sample type that was acceptable by the FDA to be offered by a laboratory developed test in the United States. The immediate response of the CDC and FDA, providing support to commercial laboratories and allowing accredited laboratories to develop molecular diagnostics, contributed to the rapid acceleration of mpox diagnostics. However, the initial validation and implementation challenged the laboratories, primarily due to the infrequency of control materials and unavailability of clinical samples.

A collaborative model where resourced industrial partners develop a fully automated assay and support an analytical and *in silico* validation and work closely with clinical and reference laboratories to provide a real-life assay’s evaluation and clinical validation is a practical solution for accelerating the development of high-quality diagnostics. In this study, we report the multi-site evaluation of the NeuMoDx MPXV assay by three sites: the Johns Hopkins University (US), UZ Gent (Belgium, Europe), and Hospital Universitario San Cecilio (Spain, Europe) after an analytical validation by Qiagen (GmbH, Hilden, Germany). The NeuMoDx is a fully automated system designed to ease the pressure on laboratories by providing a research solution with an on-demand scalable workflow. Data from the three sites showed excellent performance of the NeuMoDx MPXV assay, with an overall sensitivity of 99.21% and specificity of 96.64% for lesion swab samples. The assay is capable of detecting and differentiating clades I and II, although the infrequency of clade I samples limited the clinical evaluation of this clade.

The dual-target design of the NeuMoDx assay, where two sets of primers and probes target different regions for each clade, can increase the sensitivity of the assay and potentially reduce false-negative results due to mutations within the primer or probe binding sites. A deletion in the MPXV TNF receptor gene target was reported by the CDC in September 2022 to potentially cause false-negative results with MPXV laboratory developed tests that target that region (17). Additionally, the ability of the assay to detect and differentiate clades I and II is significant for clinical laboratories due to the biosafety implications of MPXV clade I, particularly after the reported outbreaks of clade I in the Democratic Republic of the Congo in 2023 and first clusters of suspected sexual transmission of this clade. Even though, the non-variola orthopoxvirus and the clade II MPXV are excluded from select agents list, MPXV clade I is considered a select agent (18). The ability to exclude MPXV clade I facilitates the institutions’ compliance with HHS select agent regulations and allows laboratories to be prepared for any future outbreaks.

The full automation of the NeuMoDx MPXV assay improves the workflow in the laboratory and drastically reduces the hands-on time. False positive results likely related to cross-contamination or processing errors, particularly due to the high viral load of the mpox skin lesions were reported and had significant consequences (19). Full automation reduces laboratory errors, particularly when high testing volumes are encountered.

The limitations of this study include the small cohort of MPXV clade I tested samples and the lack of internal human gene that controls for the sufficiency of sample collections. The *in silico* analysis showed excellent inclusivity for clade I genomes, however, some cross-reactivity of clade I primers/probe sets might be expected with other orthopoxviruses including smallpox, cowpox, camelpox, and vaccinia. False-negative results due to improper sample collection, as was reported recently (20), could only be identified if an internal control human gene is used as a part of the reaction. Assays for diagnosing skin lesions largely do not include such controls though and continuous laboratory monitoring and education have been instrumental in assuring best-practice sample collections.
